# Case of Extirpation of the Parotid Gland

**Published:** 1826-07

**Authors:** 


### EXTIRPATION OF AN EXTRAORDINARY LARGE SCIRRHOUS PAROTID GLAND. BY DR. PRIEGER OF KREUZNACH.

This dangerous, and by some persons deemed impracticable operation, has been performed five times in Germany within the last five years. Dr. Prieger published his first case in which he performed this operation on a woman, in Vol. II. of Graefe and Walther's Journal: the patient was, at the time of writing the present history, in good health, and was the mother of a healthy child. Professor WEINHOLD of Halle, is said to have extracted the parotid gland, partially and completely, three times; the first time a large portion of the indurated gland was removed by applying a ligature around its base: the second extirpation which was only partial was performed by the knife, the trunk of the carotid was laid bare, and the whole of the upper part of the gland dissected out. Professor WALTHER of Bonn, has also described a case which was considered at the time to have been a scirrhous parotid which he removed. He has since, however, changed his opinion, and acknowledges that it was only a sarcomatous tumour which grew over the parotid. The same doubts do not, however, apply to the following case of Dr. Prieger's, which we find in the second number of *Rust's Magazine* of 1825.

Juliane Heimen of Mittelreulenbach, in Saxe-Cobourg, 43 years of age, of a middle stature and weak constitution, the mother of eight children, escaped all the dangers incident to the diseases of children, and enjoyed, even to the period of her becoming pregnant, very good health. When she was about thirty-three, she was confined to her bed for some weeks with a nervous fever; after her recovery from which, without any known cause, she was attacked in the night with a violent pain in the right side of the cheek and neck, so sudden, that she was obliged to have her head supported by her husband. Without using any medicine, this pain gradually diminished, but how long after, the patient could not exactly recollect. There was no swelling of the part at the time, but the patient perceived afterwards a small hard tumour in the lower part of the gland just opposite the ear; when it was first discovered, it was not larger than a pin's head, but during nine years it regularly increased to the size now about to be described. The swelling was sought to be dispersed by plaisters and various applications, but without avail.

The tumour was hard, uneven, the prominences on it sensitive and slightly reddened, somewhat tuberculated and immoveable. It commenced on the outer side of the right ear, gradually ascended toward the zygomatic process of the superior maxillary bone, protruded over it, extended itself toward the lower eye-lid, from which it was only half an inch distant, and then descended toward the nose and corner of the mouth. The eye was always in a state of epiphora as if the lachrymal canal was stopped up. The nose and angle of the mouth were, from the weight of the tumour, drawn downwards, so that at last there was almost a constant flow of saliva over the chin. The swelling extended over the right side of the chin toward the larynx, which was pressed very much toward the left side; the face was so much turned to the left side by the weight and size of the tumour, that the chin projected over the second left rib. The tumour as it continued to increase, extended backwards and downwards over the mastoid process, pushed up the lobe of the ear, and caused it to project very considerably forwards. The breadth of the lower part of the swelling measured eight inches, and on its surface many blood-vessels were visible.

The patient was very much emaciated, and bore the marks of much anxiety and pain in her countenance: the sleep was very disturbed, the pain in the head frequent, violent, and pulsating; she was obliged to lie on the left side, and complained of great pain in the throat when she attempted to swallow. The pulse, was in general regular, and menstruation had not ceased. The middle of the tumour first began to be painful and red, and the patient every day became more cachectic, so that no doubt remained that the swelling would go over into a *cancer apertus*. The operation was performed on the second day of Dr. P's seeing the patient.

"*Operation*. On the 7th of September, 1824, the operation was performed as follows. The patient was placed on a mattrass with her head supported, and turned toward the left side, and now, taking the tumour in my left hand, and drawing it upwards as much as possible with a convex bistoury, I commenced a semicircular incision from below upwards, the incision commencing near the larynx, and its convexity turned toward the chin, the dissection was continued from within outwards, and then from above downwards, so that the greatest part of this enormous mass was separated without much difficulty, with the exception of about an inch of its structure, which was very much hardened, and lying between the angle of the lower jaw and the processus mastoideus, the point in which the swelling commenced. This part was separated with the greatest care, and in the bottom of the wound the *carotis facialis* was distinctly seen pulsating. During the operation, the patient did not complain of much pain, except when the branches of the portio dura, and the maxillaris inferior were cut through; it was then very severe. The quantity of blood lost was very considerable, amounting to twenty ounces, although the vessels were secured as speedily as possible. Eleven arteries, branches of the temporal, transversus faciei and carotis facialis required to be tied. By the most careful and repeated dissection which so large an extent of wound readily allowed, all the parotid was removed: not a trace of the gland itself, or of the glandula accessoria was allowed to remain. The wound was dressed by first bringing the edges together by sutures, over them were laid straps of adhesive plaster, then a compress, and the whole secured with an appropriate bandage. The patient was kept quiet in a dark chamber, and allowed nothing but a little gruel.

The tumour after it had been for some time macerated in water, and the blood drawn out, weighed *three pounds and a half* apothecaries' weight—it was on all sides uneven, tumulated, and throughout of a very fine consistence. With the exception of a few unimportant accidents, as a little difficulty of swallowing, which continued only a few days, and a little cough, which was readily quieted by the ordinary remedies, nothing peculiar happened during the process of cure. On the 10th of September, the third day after the operation, some of the sutures were removed, as the adhesive plaster was found sufficient to hold the edges together. On the 12th, all the sutures were taken out. The ligatures began to separate on the 11th, and on the 20th, they were all withdrawn. The granulating process proceeded most rapidly, every day there was an evident improvement, and on the 1st of October, the patient left the hospital perfectly cured with a cicatrix about three lines broad in some places, and set out on foot to make a journey of eight hours (almost 20 English miles) to her native home.

The perusal of this case shows that the operator is a bold surgeon, and a good anatomist. It is to be regretted, that he has not described the structure of the tumour more accurately, as from what he has said of it, it is impossible to judge whether it was scirrhous or not. The rapidity of the cure is almost as astonishing as the extent and progress of the disease. Richerand, Boyer, Richter, and Mr. Allan Burns, have all contended, that such an operation was impossible; the enterprizing spirit of modern surgeons has shown the contrary, and the adopted motto appears to be *nil desperandum.*

